# An anomaly detection approach to identify chronic brain infarcts on MRI

**DOI:** 10.1038/s41598-021-87013-4

**Published:** 2021-04-08

**Authors:** Kees M. van Hespen, Jaco J. M. Zwanenburg, Jan W. Dankbaar, Mirjam I. Geerlings, Jeroen Hendrikse, Hugo J. Kuijf

**Affiliations:** 1 Center for Image Sciences, University Medical Center Utrecht, Heidelberglaan 100, Postbox 85500, 3584 CX Utrecht, The Netherlands; 2 Department of Radiology, UMC Utrecht, Heidelberglaan 100, 3584 CX Utrecht, The Netherlands; 3 Julius Center for Health Sciences and Primary Care, UMC Utrecht, Heidelberglaan 100, 3584 CX Utrecht, The Netherlands; 4 Image Sciences Institute, UMC Utrecht, Heidelberglaan 100, 3584 CX Utrecht, The Netherlands

**Keywords:** Brain imaging, Magnetic resonance imaging, Neurological disorders, Image processing, Machine learning, Cerebrovascular disorders

## Abstract

The performance of current machine learning methods to detect heterogeneous pathology is limited by the quantity and quality of pathology in medical images. A possible solution is anomaly detection; an approach that can detect all abnormalities by learning how ‘normal’ tissue looks like. In this work, we propose an anomaly detection method using a neural network architecture for the detection of chronic brain infarcts on brain MR images. The neural network was trained to learn the visual appearance of normal appearing brains of 697 patients. We evaluated its performance on the detection of chronic brain infarcts in 225 patients, which were previously labeled. Our proposed method detected 374 chronic brain infarcts (68% of the total amount of brain infarcts) which represented 97.5% of the total infarct volume. Additionally, 26 new brain infarcts were identified that were originally missed by the radiologist during radiological reading. Our proposed method also detected white matter hyperintensities, anomalous calcifications, and imaging artefacts. This work shows that anomaly detection is a powerful approach for the detection of multiple brain abnormalities, and can potentially be used to improve the radiological workflow efficiency by guiding radiologists to brain anomalies which otherwise remain unnoticed.

## Introduction

In clinical practice, radiologists acquire and assess magnetic resonance (MR) images of the brain for the diagnosis of various brain pathologies. Unfortunately, the process of reading brain MR images is laborious and observer dependent^1–5^. To reduce observer dependence, and to improve workflow efficiency and diagnostic accuracy, automated (machine learning/‘artificial intelligence’) methods have been proposed to assist the radiologist^6–13^. A common drawback of these methods is their ‘point solution’ design, in which they are focused on a specific type of brain pathology. Furthermore, the performance of supervised machine learning based solutions is dependent on the quantity and quality of available examples of pathology. In, for example, cerebral small vessel disease, the development of such solutions is challenging, because the parenchymal damage is heterogeneous in image contrast, morphology, and size^14,15^.

A solution that breaks with this conventional approach is anomaly detection: a machine learning approach that can identify all anomalies solely based on features that describe normal data. Because the features of possible anomalies were not learnt, they stand out from the ordinary, and can subsequently be detected. Anomaly detection methods are particularly useful when there is an interest in the detection of anomalous events, but their manifestation is unknown a priori and their occurrence is limited^16,17^. Examples of applications include credit card fraud detection^18^, IT intrusion detection^19^, monitoring of aerospace engines during flight^20^, heart monitoring^21^, detection of illegal objects in airport luggage^22^, or the detection of faulty semiconductor wafers^23^.

In medical imaging, variational autoencoders and generative adversarial networks have been proposed for anomaly detection tasks. Schlegl et al. have developed a generative adversarial network architecture for the detection of abnormalities on optical coherence tomography images^24,25^. For brain MRI, models have been developed for the detection of tumor tissue^26–28^, white matter hyperintensities^29,30^, multiple sclerosis lesions^31,32^, and acute brain infarcts^28^.

One of the manifestations of cerebral small vessel disease are chronic brain infarcts, including cortical, subcortical, and lacunar infarcts; each with a different appearance on MRI^14,15^. Identification of these infarcts is important, because their occurrence is associated with vascular dementia, Alzheimer’s disease, and overall cognitive decline^33,34^. Because of the heterogeneity in appearance of chronic brain infarcts, location and morphology on MR imaging, anomaly detection would be a possible solution for the identification of these infarcts.

In this study, we constructed an anomaly detection method using a neural network architecture for the detection of chronic brain infarcts from MRI.

## Materials and methods

### MR acquisition

**Figure 1:**
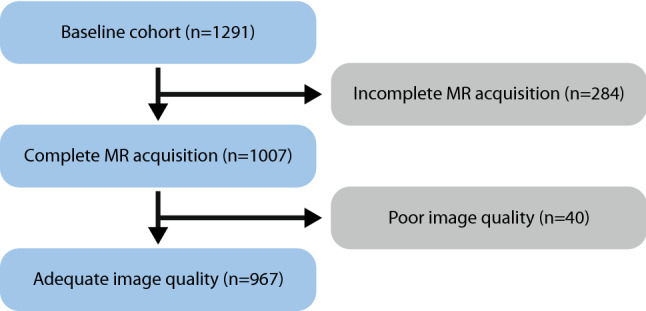
Flowchart showing exclusion of patients in the current study. In total 324 out of 1291 patients were excluded from the current study because of an incomplete MR acquisition (284), where one of two acquired MR images were missing, and because of poor image quality (40).

**Figure 2:**
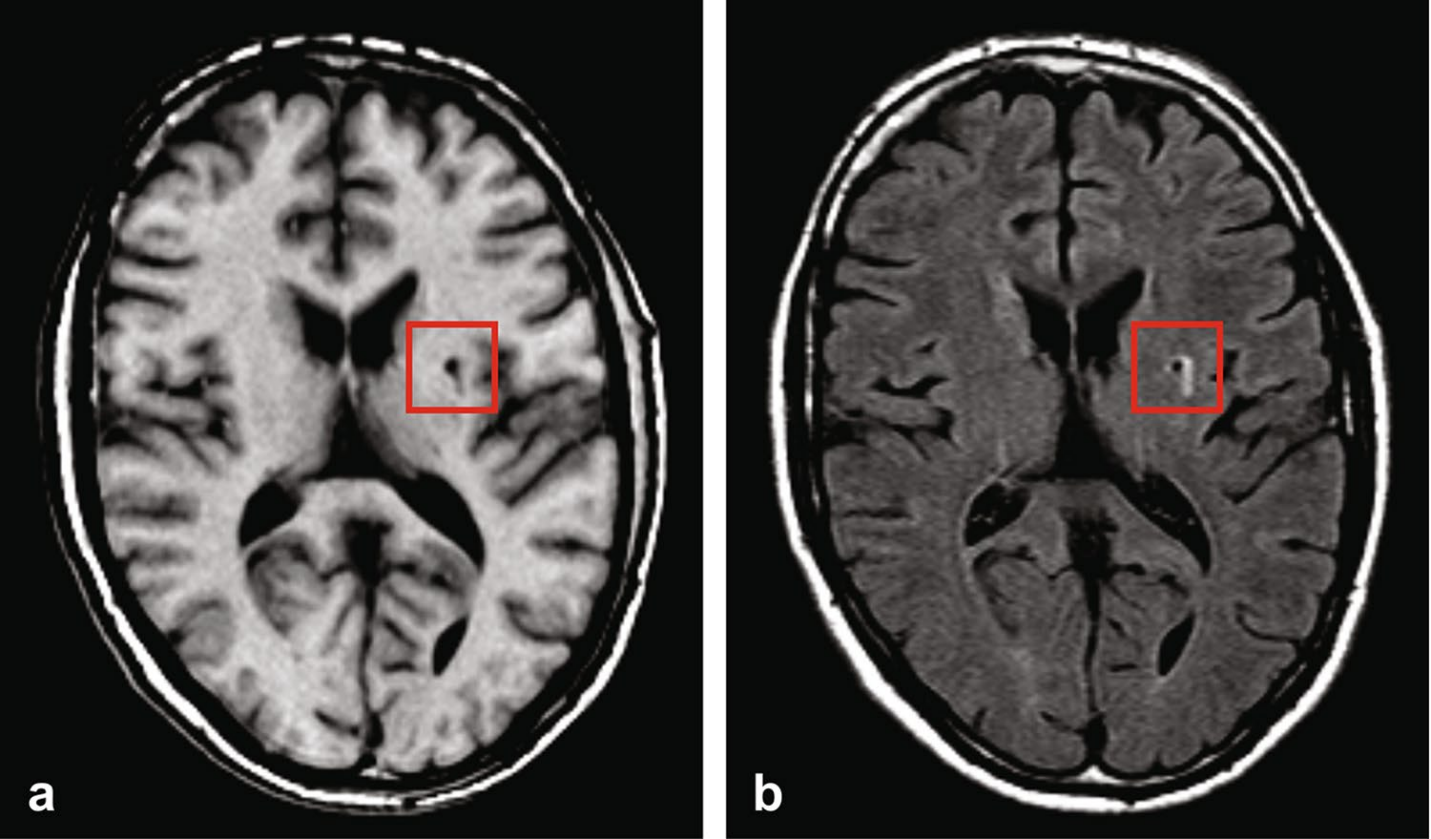
Example transversal image slice of the T1-weighted (**a**) and T2-FLAIR (**b**) acquisitions. A brain infarct can be observed in the right hemisphere next to the basal ganglia, in the red square, as the hypointense region in the T1-weighted image (**a**) and the hypointense region with hyperintense ring in the T2-FLAIR image (**b**).

In this retrospective study, we used MR image data from the SMART-MR study^35^, a prospective study on the determinants and course of brain changes on MRI, where all eligible patients that were newly referred to our hospital with manifestations of coronary artery disease, cerebrovascular disease, peripheral arterial disease or an abdominal aortic aneurysm were included after acquiring written informed consent. This study was conducted in accordance with national guidelines and regulations, and has been approved by the University Medical Center Utrecht Medical Ethics Review Committee (METC). In total 967 patients, including 270 patients with brain infarcts (see Table 1 for patient demographics), were included in the current study, see Fig. 1 for exclusion criteria. The imaging data was acquired at 1.5 T (Gyroscan ACS-NT, Philips, Best, the Netherlands), and consisted of a T1-weighted gradient-echo sequence (repetition time (TR) = 235 ms; echo time (TE) = 2 ms), and a T2-weighted fluid-attenuated inversion recovery (T2-FLAIR) sequence (TR = 6000 ms; TE = 100 ms; inversion time: 2000 ms) (example given in Fig. 2). Both MRI sequences had a reconstructed resolution of 0.9 × 0.9 × 4.0 mm^3^, consisted of 38 contiguous transversal slices, and were coregistered^35^. A reasonable request for access to the image data can be send to Mirjam Geerlings (see author list). The code that supports the findings of this study is publicly available from Bitbucket (https://bitbucket.org/KeesvanHespen/lesion_detection).

**Table 1:** Demographics of the patient groups with and without brain infarct, used for the training/training-validation, and validation/test sets, respectively.

Characteristics	Patients without brain infarcts	Patients with brain infarcts
No. of patients	697	270
Age (years)	57 (± 10)	62 (± 10)
Male sex	550	213

All chronic brain infarcts -including cortical infarcts, lacunar infarcts, large subcortical infarct, and infratentorial infarcts- in these images, have been manually delineated by a neuroradiologist with more than 30 years of experience, as described by Geerlings et al.^35^ in more detail.

### Image preprocessing

The images of both acquisitions were preprocessed by applying N4 bias field correction^36^. Additionally, image intensities were normalized such that the 5th percentile of pixel values within an available brain mask was set to zero, and the 95th percentile was set to one.

Two dimensional image patches (smaller subimages of the original image) were sampled within an available brain mask^37^ at corresponding locations on both acquisitions for four datasets; a training set, a training-validation set, a validation set, and a test set. For the training set, one million transversal image patches (15 × 15 voxels) were sampled from images of all but ten patients without brain infarcts. The remaining ten patients without brain infarcts were used for the training-validation set, which was used to assess potential overfitting of the network on the training set. For the training-validation set, 100,000 image patches were randomly sampled. The patches for the training and training-validation set were augmented at each training epoch, by performing random horizontal and vertical flips of the image patches.

The performance of our method on the detection of brain infarcts was evaluated on the validation and test set. The validation set, which was used to evaluate several model design choices consisted of 45 randomly selected patients with brain infarcts. The remainder (225) was included in the test set, on which the final network performance was evaluated. In the validation and test sets, 93, and 553 brain infarcts were present, with a median volume of 0.4 ml (range 0.072–282 ml) and 0.44 ml (range 0.036–156 ml), respectively. For these sets, the entire brain was sampled, using a stride of four voxels.

### Network architecture

**Figure 3:**
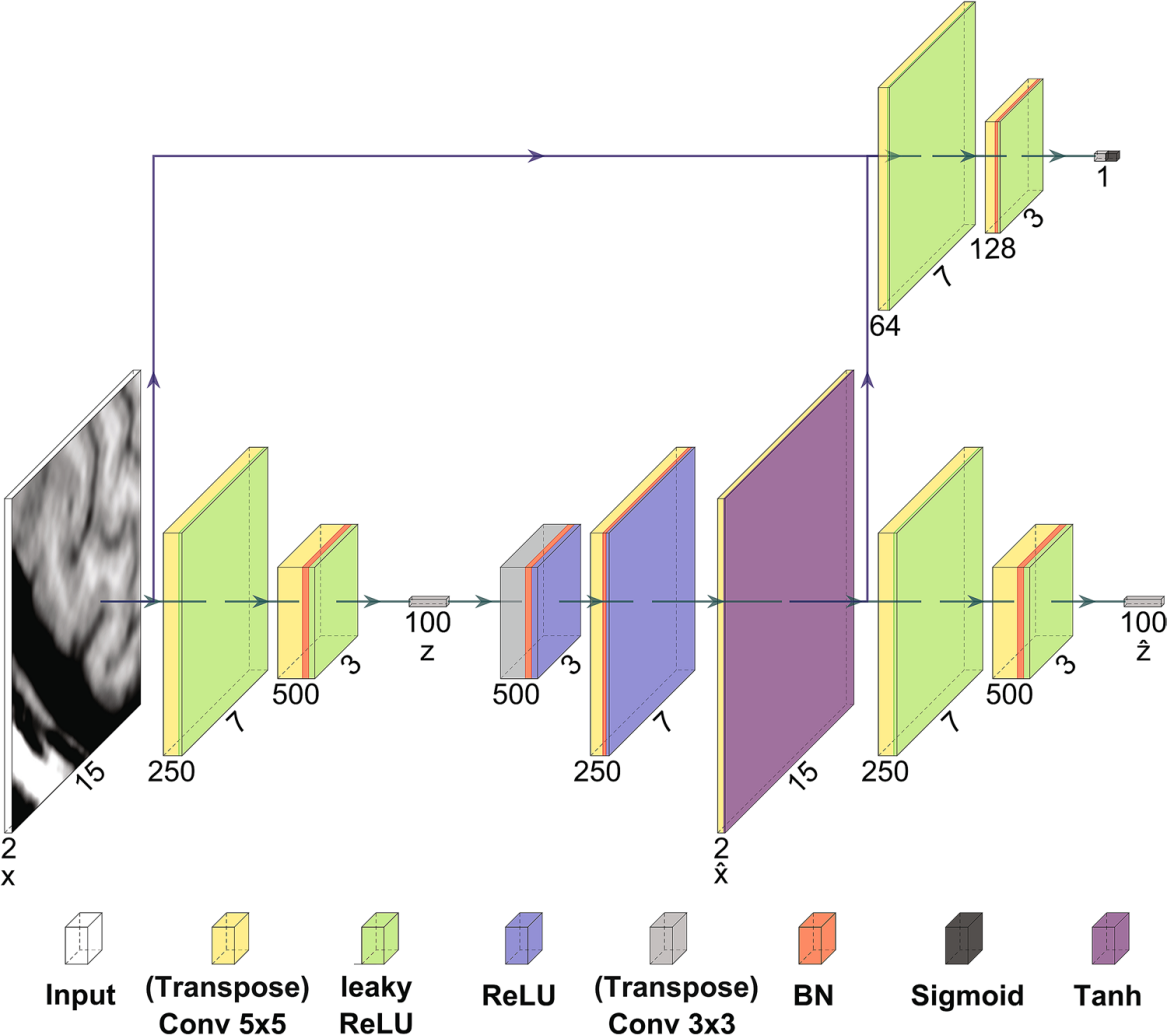
Neural network architecture, visualized using adapted software of PlotNeuralNet^39^. The tensor size after each operation is given by the numbers around each box. The input images are encoded into a latent space ${\varvec{z}}$. From the latent space a reconstruction $\widehat{{\varvec{x}}}$ of the input ${\varvec{x}}$ is created. A final encoding of $\widehat{{\varvec{x}}}$ to $\widehat{{\varvec{z}}}$ is computed. A discriminator, featured in the top of the image, is fed with images ${\varvec{x}}$ and $\widehat{{\varvec{x}}}$, where the output of the first to last layer is used for the calculation of the adversarial loss ${{\varvec{L}}}_{{\varvec{a}}{\varvec{d}}{\varvec{v}}}$. Similarly, a reconstruction loss ${{\varvec{L}}}_{{\varvec{c}}{\varvec{o}}{\varvec{n}}}$ is calculated as the mean difference between ${\varvec{x}}$ and $\widehat{{\varvec{x}}}$, and an encoding loss ${{\varvec{L}}}_{{\varvec{e}}{\varvec{n}}{\varvec{c}}}$ is calculated as the L2 loss between ${\varvec{z}}$ and $\widehat{{\varvec{z}}}$. The (transposed) conv 5 × 5 layers are applied with a stride of 2 and padding of 1, and are in almost all steps followed by batch normalization (BN). The conv 3 × 3 layer is applied with a stride of 1 and padding equal to 0. The leaky rectified linear unit (ReLU) activation has a negative slope equal to 0.2.

We implemented a neural network architecture based on the GANomaly architecture in PyTorch v1.1.0^22,38^ which ran on the GPU of a standard workstation (Intel Xeon E-1650v3, 32gb RAM, Nvidia Titan Xp). The neural network (Fig. 3) consisted of a generator (bottom half) and discriminator (top half). The input of the network features two input channels, for both the T1-weighted and the T2-FLAIR image patches. The generator and discriminator consisted of encoder and decoder parts, that each contained three sequential (transposed) convolutional layers, interleaved with (leaky) rectified linear unit (ReLU) activation and batch normalization. The generator was trained to encode the input image patches $x$ into latent representations: $z$ and $\widehat{z}$. Additionally, the generator was trained to realistically reconstruct the input images from the latent vector $z$ into the reconstructed image $\widehat{x}$. The discriminator was used to help the generator create realistic reconstructions $\widehat{x}$. The latent representations $z$ and $\widehat{z}$ were used to calculate an anomaly score per image patch.

During training of the generator, three error terms were minimized. First, the reconstruction error was computed as the mean difference between the input image and reconstructed image (${L}_{con}={\Vert x-\widehat{x}\Vert }_{1})$. Second, the encoding error was given by the L2 loss between the latent space vectors $z$ and $\widehat{z}$ (${L}_{enc}={\Vert z-\widehat{z}\Vert }_{2}$). Last, the adversarial loss was computed as the L2 loss between the features from first to last layer of the discriminator, given the input image and reconstructed image (${L}_{adv}={\Vert f(x)-f(\widehat{x})\Vert }_{2}$). To balance the optimization of the network, the generator loss was computed as a weighted combination of the aforementioned losses, with weights of 70, 10, and 1 for ${L}_{con}$, ${L}_{enc}$, and ${L}_{adv}$ respectively.

For the discriminator, two error terms were minimized during training^40^. The first term, is given by the binary cross entropy of the input images and the label of the reconstructed images, and the second term is given by the binary cross entropy of the reconstructed images and the label of the input images. To prevent vanishing gradients in the discriminator, a soft labeling method was chosen, where labels for the reconstructed and input images were uniformly chosen between 0 and 0.2, and between 0.8 and 1, respectively.

The network was trained using the training image patches, which were fed to the network in minibatches of size 64. A learning rate of 0.001 was used, with Adam as optimizer^41^. Training continued until the generator loss on the training-validation set did not decrease any further for ten epochs. The network weights, for the epoch with the lowest generator loss were used for testing.

### Anomaly scoring

An anomaly score was calculated per image patch as the modified Z-score, a measure of how many median absolute deviations a value lies away from a median value. During training, a median and median absolute deviation were calculated per element of the difference vector $z-\widehat{z}$, over all training image patches. These values were used to calculate the modified Z-score for all of the difference vector elements of the validation/test image patches. The anomaly score for each image patch was calculated by taking the Nth percentile of modified Z-score values over all vector elements. The value of the percentile was determined in the first experiment.

The anomaly scores over all image patches were projected back onto the original brain image, where areas with an anomaly score larger than 3 were flagged as suspected anomalies. Spurious activation, where only a single isolated patch had an anomaly score larger than 3, were filtered out from the final result.

### Experiments

#### Latent vector size and anomaly score calculation

We investigated the effect of the size of the latent vectors $z$ and $\widehat{z}$ (Fig. 3), and the effect of the anomaly score calculation on the detection of brain infarcts. We trained several neural network instances with a varying latent vector size, namely: 50, 75, 100, 150, 200, 300, and 400 vector elements. Additionally, we varied the used percentile *N* for the anomaly score calculation between 10 and 75, in steps of 5 percentage points. For both parameters, we computed the sensitivity and the average number of suspected anomalies per image, as well as the volume fraction of the detected brain infarcts compared to the total brain infarct volume.

#### Suspected anomaly classification

We used the optimal parameters to evaluate the performance of our proposed method on the test set. We computed the sensitivity and the average number of suspected anomalies per image. In addition, we analyzed the origin of the remaining suspected anomalies, where a neuroradiologist with more than 10 years of experience (JWD), classified these suspected anomalies as one of seven classes, namely: normal tissue, unannotated brain infarct, white matter hyperintensity, blood vessel, calcification, bone and image artefact.

#### Missed brain infarcts

We investigated why our proposed method missed some brain infarcts by evaluating the volume and location of these missed brain infarcts. Additionally, we performed a nearest neighbor analysis, in which we analyzed which training image patches were similar to the test image patches with missed brain infarcts.

## Results

### Latent vector size and anomaly score calculation

**Figure 4:**
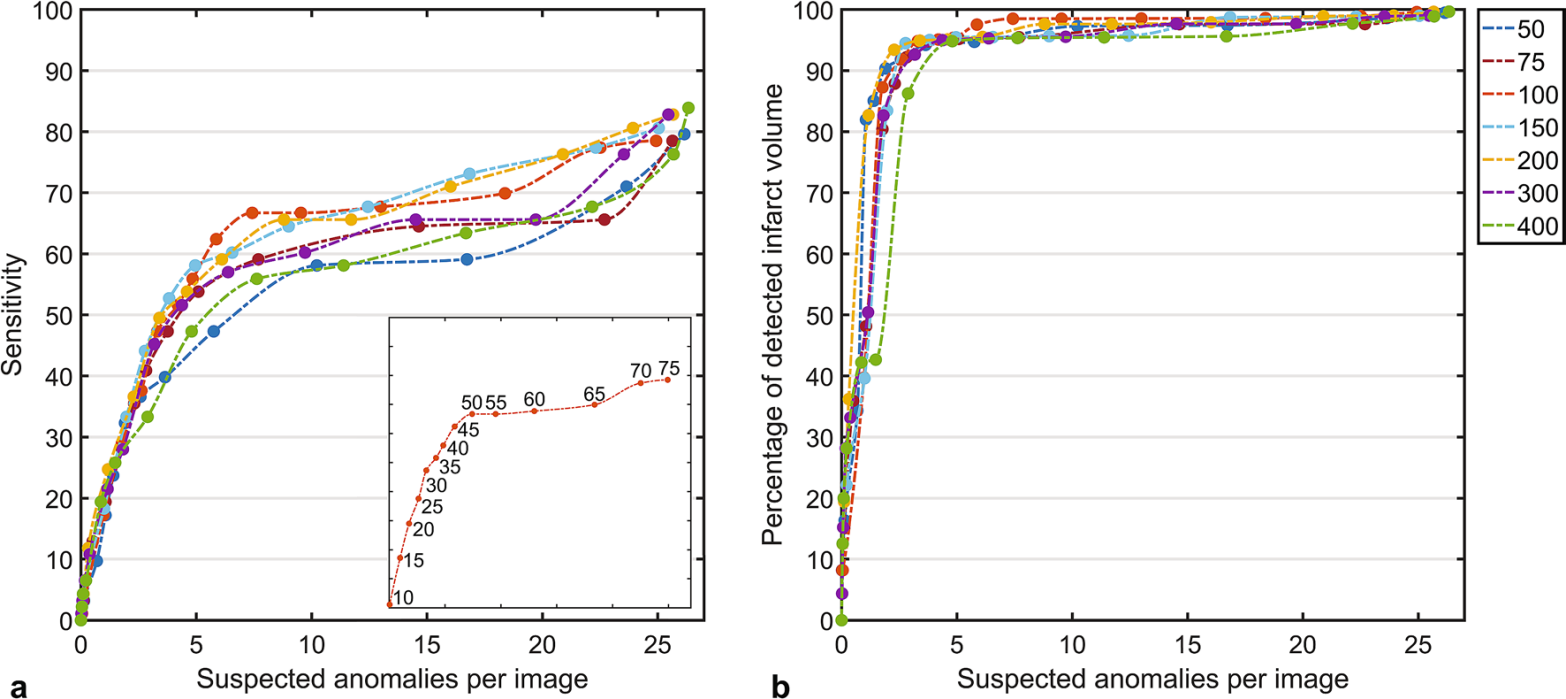
Brain infarct detection performance for various latent vector sizes and percentile values *N,* used in the anomaly score calculation. (**a**) Free Response Operator Curve for all latent vector sizes and (**b**) detected brain infarct volume to total brain infarct volume ratio (given in color). The used percentile ranged between 10 and 75, where for all lines the first datapoint on the left corresponds to 10th percentile, and the right corresponds to the 75th percentile. The inset in (**a**) shows the used percentiles for a latent vector size of 100. The highest sensitivity for the lowest number of suspected anomalies per image (68% and 6) is given by a latent vector size of 100 and a percentile of 50.

Based on the validation set, a latent vector size of 100 showed the highest sensitivity and detected brain infarct volume fraction for the same number of suspected anomalies over almost the entire range, compared to all other latent vector sizes (Fig. 4). Similarly, using the 50th percentile yielded an optimal tradeoff between sensitivity and fraction of detected brain infarct volume against the number of suspected anomalies. Given our validation dataset, the optimal parameters for the detection of brain infarcts include the use of the 50th percentile with a latent vector size of 100.

### Suspected anomaly classification

**Figure 5:**
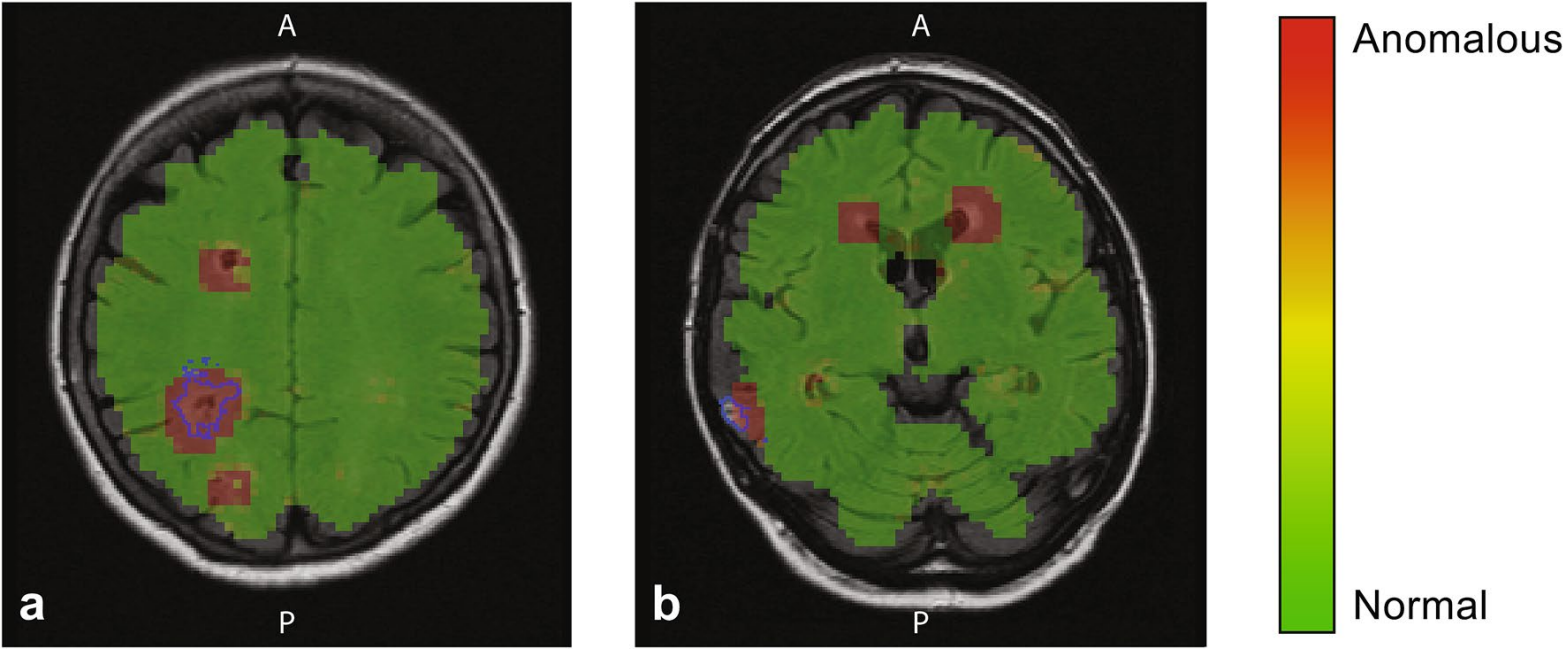
Anomaly score overlay maps on transversal image slices (T2-FLAIR images). A red color indicates an anomaly score ≥ 3, an anomalous location, where green and yellow an anomaly score < 3, normal tissue. The manually annotated brain infarcts are given by the blue outlines. An unannotated brain infarct located in a cranial slice can be observed in (**a**), anterior (A) of the annotated brain infarct. The anomaly at the posterior (P) side of the brain infarct in (**a**) is only delineated in a subsequent image slice. (**b**) Detected cortical brain infarct, and two other suspected anomalous locations, caused by white matter hyperintensities at the horn of the ventricles.

We used the optimal parameters to evaluate the performance of our proposed method on the test set, on which our proposed method found on average nine suspected anomalies per image (total: 1953, examples are given in Fig. 5). In total, 374 out of 553 brain infarcts were detected by our model (sensitivity: 68%), representing 19.2% of all suspected anomalies (Table 2). These detected brain infarcts represented 97.5% of the total brain infarct volume. Eight hundred and sixty-five (44.3%) suspected anomalies were caused by white matter hyperintensities. Image artefacts, e.g. due to patient motion, attributed to 115 (5.9%) of all suspected anomalies. Normal healthy tissue was accountable for 563 (28.8%) of all suspected anomalies. In most cases, these normal tissue false positives were located at tissue—cerebrospinal fluid boundaries. Most interestingly, 26 (1.3%) of all suspected anomalies corresponded to unannotated brain infarcts, which were oftentimes located in the cerebellum and the most cranial image slices.

**Table 2:** Suspected anomaly categories in the 225 test subjects (Total: 1953. On average: 9 suspected anomalies per image).

Suspected anomaly category	Count (%)
White matter hyperintensity	864 (44.3)
Normal tissue	563 (28.8)
Annotated brain infarcts	374 (19.2)
Image artefact	115 (5.9)
Unannotated brain infarct	26 (1.3)
Blood vessel	6 (0.3)
Calcification	2 (0.1)
Bone	2 (0.1)

The suspected anomalies were categorized in eight categories, where the categorization was performed manually by a trained radiologist, except for the ‘annotated brain infarcts’ (these annotations were already available from the used image dataset).

### Missed brain infarcts

**Figure 6:**
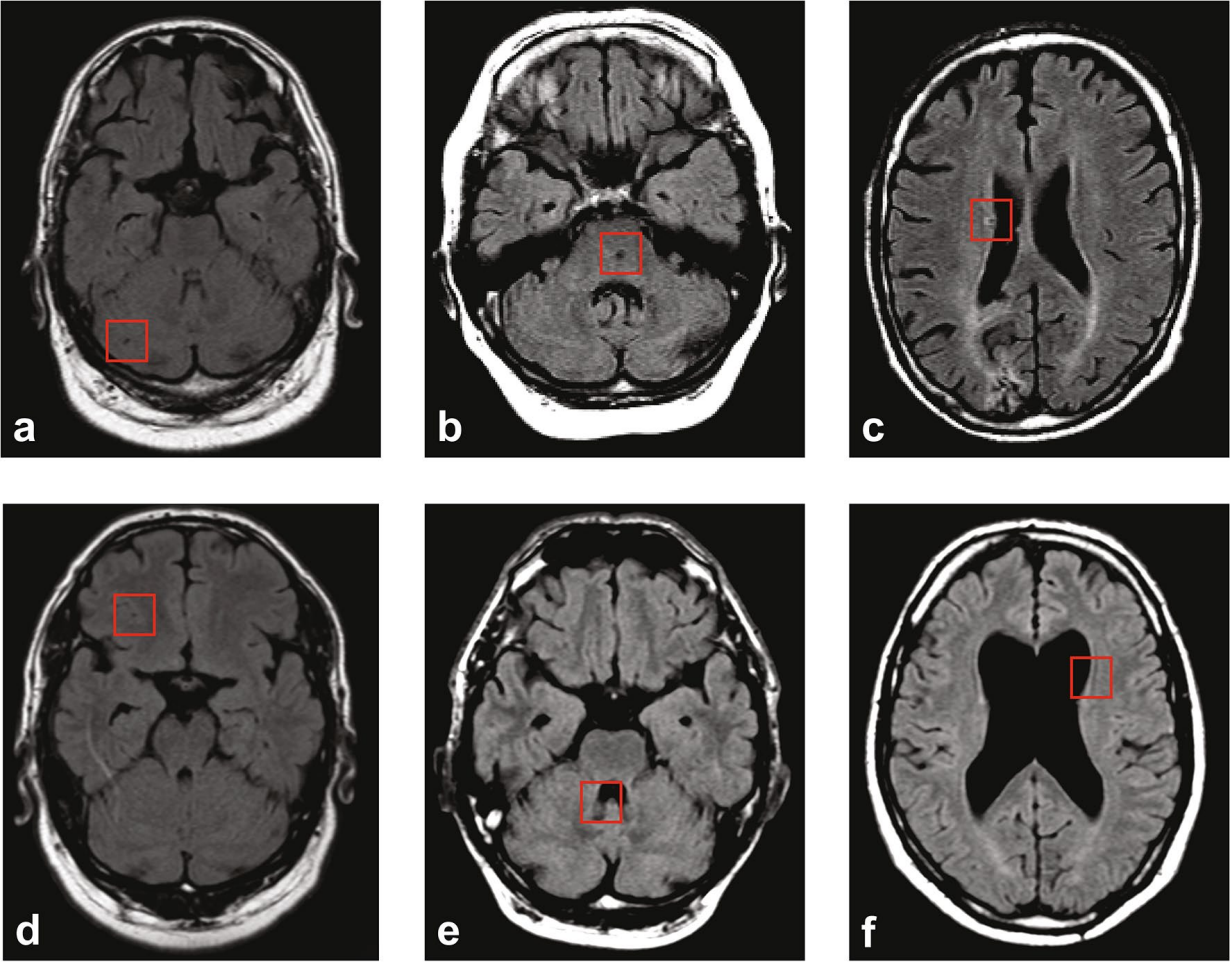
Comparison between missed brain infarcts and nearest neighbor locations in training images. (**a**–**c**) Missed brain infarcts indicated by the red square in the cerebellum, brainstem and cerebrum, respectively, on transversal T2-FLAIR images. (**d**–**f**) the corresponding nearest neighbor locations in training data matching (**a**–**c**) respectively. The square in (**d**) contains a part of a normal sulcus that has a similar appearance as the missed brain infarct in the cerebellum in (**b**).

We performed an additional experiment to better understand why our proposed method missed 179 small brain infarcts (2.5% of the total brain infarct volume). Volume analysis revealed that the missed brain infarcts were in almost all cases smaller than 1 ml (median volume = 0.23 ml). Almost half of the missed brain infarcts (75) were located near the ventricles, at the level of the basal ganglia. Twenty-three missed brain infarcts were present in the cerebellum and 26 in the brain stem. The remaining 55 brain infarcts were located in the cerebral cortex. Automated analysis, where we used a nearest neighbor algorithm to determine which training image patches were similar to the missed brain infarct image patches, revealed that missed brain infarct image patches were oftentimes closely related to training image patches that contained sulci or tissue—cerebrospinal fluid boundaries (see Fig. 6). Furthermore, missed brain infarcts in the brainstem were mostly linked to training image patches in the cortical region, and image patches in the brainstem that border on the cerebrospinal fluid looked similar to cortical image patches with cerebrospinal fluid.

## Discussion

In this paper, we proposed an anomaly detection method for the detection of chronic brain infarcts on brain MR images. Our proposed method detected brain infarcts that accounted for 97.5% of the total brain infarct volume of 225 patients. The missed brain infarcts had in most cases a volume smaller than 1 ml, and were mostly located in the brain stem, and cerebellum, and next to the ventricles. White matter hyperintensities, anomalous calcifications, and imaging artefacts accounted for 44.3%, 0.1%, and 5.9% of suspected anomalies, respectively. Additionally, our proposed method identified additional brain infarcts, which were previously missed by the radiologist during radiological reading (1.3% of all suspected anomalies).

Our proposed method properly evaluated regions that are more easily overlooked by a trained radiologist, given the additional brain infarcts that our method found. The percentage of unannotated brain infarcts that our proposed method found (5% of all brain infarcts) is in accordance with literature, which suggest that reading errors occur in 3–5% of the cases in day-to-day radiological practice. These errors can occur because of an inattentional bias, where radiologists are focused on the center of an image, while overlooking findings at the edges of the acquired image^3^. As workload in radiology is ever increasing due to a larger load of radiological images to be assessed, it is even more easy to overlook brain pathologies^42^. Our presented automated analysis method can potentially alleviate part of this assessment by suggesting anomalous areas for the radiologist to look at, or guiding the radiologist to potentially overlooked brain areas.

Besides suggesting anomalous areas during assessment of the scans by a radiologist, anomaly detection can also be used during image acquisition. In case of an important suspected pathology, this information can then instantly be used to make changes to the acquisition protocol by relocating the field of view or by adding an acquisition that is important for subsequent analysis of the suspected pathology.

Cerebral small vessel disease is a disease with multiple manifestations on MR images. Our proposed method has shown that it can detect at least two of these manifestations, namely chronic brain infarcts -including cortical infarcts, lacunar infarcts, large subcortical infarct, and infratentorial infarcts- and anomalous white matter hyperintensities. This is in contrast to other methods^6,43^, that are only trained for the detection of a single homogeneous type of brain pathology. Similarly, other heterogeneous brain pathologies such as brain tumors, or other manifestations of cerebral small vessel disease can also likely be detected using anomaly detection.

Anomaly detection, which is already used for several years in various fields, such as banking, aerospace, IT, and the manufacturing industry, can also be further explored in the medical imaging field. Potential other applications of anomaly detection include the detection of lung nodules on chest CT images, anomalous regions in the retina, breast cancer in mammograms, calcifications in breast MRI, liver tumor metastasis, or for the detection of areas with low fiber tract integrity in diffusion tensor imaging^25,44–47^. Also for the detection of artefacts in MR spectroscopy or the detection of motion artefacts in MR images, anomaly detection can potentially be beneficial^48,49^. For example, by analyzing the acquired k-space data on motion artefacts during patient scanning, a decision can be made more quickly to redo (parts of) the acquisition.

In future work, the use of 2.5D or 3D contextual information can potentially mitigate problems related to the interpretation of small brain infarcts. This is preferably done with MR images with an isotropic voxel size. This approach would mimic the behavior of human readers who also use contextual information, by scrolling through images, when reading an image. Additional to the performance improvement on small brain infarcts, false positive detections of normal tissue can potentially be mitigated by adding contextual information.

## Limitations

Our method has several limitations. First, its performance on brain infarcts smaller than 1 ml is limited. The detection of small lesions is a common problem that is also present in other medical image analysis applications^50–55^. Ghafoorian et al. have aimed to improve the detection of small lesions by splitting image processing into pathways finetuned to large and small lesions^56^. For our approach, other network architectures might be investigated. Our current architecture seems to predominantly find large anomalies with a relatively large contrast difference compared to the surrounding brain tissue. Other approaches (e.g. a recurrent convolutional neural network^51^) might be able to put more emphasis on finding smaller anomalies with a lower contrast compared to the background. Besides improvements to image analysis, changes in image acquisition may also contribute to better lesion detection. Performance of the detection of small lesions is expected to improve with more up-to-date scanning protocols, which oftentimes have a higher signal-to-noise ratio and/or higher spatial resolution. Van Veluw et al. have shown that microinfarcts can be detected at 7 T MRI^57^, and later work has shown that these small lesions can be imaged at 3 T MRI as well^58^. The current work was done on 1.5 T data with limited sensitivity for detecting small lesions.

Design choices on the neural network architecture were made based on its performance on the validation set that included some brain infarcts. This does not reflect complete anomaly detection, where anomalies should be completely unknown. However, such an approach is unfeasible in normal practice. Other anomaly detection methods have also used validation sets to tune hyperparameters^59–61^. The use of our validation dataset had no influence on the training of the network, because the network weights of the training epoch with the lowest training-validation loss were used, as opposed to using the network weights of the training epoch with the best brain infarct detection performance on the validation set.

Anomaly detection commonly does not involve classification of anomalies, but only their localization. In case classification is needed, automated methods or manual inspection should be performed after analysis by our proposed method. We envision a workflow in radiology routine where anomaly detection locates possible lesions, a secondary system classifies these lesions or labels them as ‘unknown’, and finally presents the results to a radiologist for inspection.

The method was developed and evaluated on data from a single cohort study. The performance on scans that are acquired on other scanners, from other vendors, and on different field strengths is therefore unknown and a topic of future work. The method could be made applicable to other scanners by partial or full retraining, or by applying transfer learning techniques. In the latter case, a relatively small new dataset might be needed.

The number of false positive detections is relatively high, compared to all other detections (28.8% of all detections). However, we believe that suggesting a few healthy brain locations to the radiologist is less of a problem than missing pathology. Our model has shown its added value by suggesting infarcts that were overlooked by the radiologist.

Lastly, our training set potentially contains unannotated brain infarcts similar to the test set, in which, 5% additional brain infarcts were detected. The potential effect of abnormalities being present in the training data on the final detection performance is likely to be low. The training will be dominated by numerous image patches from normal appearing brain tissue and any abnormalities will therefore have a minimal impact on the anomaly score calculation.

In conclusion, we developed an anomaly detection model for the purpose of detecting chronic brain infarcts on MR images, where our method recovered 97.5% of the total brain infarct volume. Additionally, we showed that our proposed method also finds additional brain abnormalities, some of which were missed by the radiologist. This supports the use of anomaly detection as automated tool for computer aided image analysis.

## Data Availability

A reasonable request for access to the image data can be send to Mirjam Geerlings (see author list). The code that supports the findings of this study is publicly available from Bitbucket (https://bitbucket.org/KeesvanHespen/lesion_detection).
